# Cross-Cultural Validation of Children's Assessment of Participation and Enjoyment Portuguese Version

**DOI:** 10.3389/fped.2019.00033

**Published:** 2019-02-12

**Authors:** Fábio Vila-Nova, Raul Oliveira, Rita Cordovil

**Affiliations:** ^1^Faculdade de Motricidade Humana, Universidade de Lisboa, Lisbon, Portugal; ^2^Centro Interdisciplinar da Performance Humana, Faculdade de Motricidade Humana, Universidade de Lisboa, Lisbon, Portugal

**Keywords:** participation, leisure, children, CAPE, measure, validation, cerebral palsy

## Abstract

**Background:** Participation is a major pediatric rehabilitation goal according to The International Classification of Functioning, Disability and Health Children and Youth version (ICF-CY). ICF-based leisure participation measures for Portuguese-speaking children with cerebral palsy are currently not available. The aim of this study is to assess validity and reliability of the Portuguese (European) version of the Children's Assessment of Participation and Enjoyment (CAPE). Methods: CAPE Portuguese version was applied to 170 children with cerebral palsy (*n* = 69) and typical development (*n* = 101) aged between 8 and 18 years (mean = 12.5 years; SD = 2.91). Construct validity was assessed by using the know-groups method and the correlation between participation and quality of life. Reliability was determined by internal consistency and test-retest.

**Results:** CAPE discriminates between participation scores of children with cerebral palsy and typical development. A positive correlation was found between participation frequency and physical well-being. Internal consistency was not entirely satisfactory but comparable with that from the original CAPE study. Test-retest reliability was considered good.

**Conclusions:** CAPE Portuguese (European) version showed satisfactory validity and test-retest reliability to assess leisure participation in children with cerebral palsy and typical development aged between 8 and 18 years.

## Introduction

Participation in everyday activities contributes to the development of children with and without disabilities. In the *International Classification of Functioning, Disability and Health* model (ICF), participation is a key element and is considered the result of the interaction between body functions and structures, activity, personal, and environmental factors. Participation is defined as the “*involvement in life situations*” and reflects a social perspective of functioning (1). In the *Children and Youth* ICF version, the activities (ability to execute a task or action in a standard environment) and participation (what an individual does in his/her current environment) domain comprise a full range of life areas, such as general tasks and demands, communication, mobility, self-care, interpersonal relations, domestic life, learning and applying knowledge, major life areas and community, social and civic life, including leisure (2).

Participation in leisure has increasingly become an area of interest for research and intervention in pediatric neurological rehabilitation (3, 4). Leisure represents a significant part of a child's daily life (5), and may refer to the involvement in formal and informal extracurricular activities, such as play, sport, entertainment, learning, and religious expression (6). Through leisure, children can learn and develop skills, interact socially, have fun, achieve and fulfill a meaning for life (7). Participating in leisure activities improves their physical, psychological, emotional health and well-being (8–10). The relationship between leisure participation and quality of life has been highlighted by a previous systematic review (10).

Cerebral palsy (CP) is a chronic health condition caused by injuries in the developing brain. The impairments in neuromotor function caused by these brain injuries may be associated with cognition, communication, and neurosensory system disorders, ultimately impacting on participation (11). Previous research shows that children with CP are at risk of restricted leisure participation. Indeed, although children with CP may participate in diverse activities and achieve high enjoyment, the frequency of participation is low. Specifically, the frequency of interactions outside the family circle is low, as these children mostly participate in activities carried out at home or at a relative's house, when compared with children without disabilities (12–16). Decreased participation is also noticed during the transition from childhood to adolescence (17). Furthermore, restriction in physical activities is observed in children with CP (18), which may negatively influence motor function and physical condition as the child grows (19, 20).

Age, gender, functional ability, and family interests have been identified as determinants of leisure participation (21). Furthermore, a number of studies reveal that significant differences in leisure participation may exist between countries. Ullenhag et al. (22) found variations in diversity and frequency of leisure participation between Sweden, Norway, and the Netherlands in children with disabilities, and the country of residence was the strongest predictor of variance in all the examined activities practiced on a regular basis. Michelsen et al. (23) also identified differences in participation among school-aged children with CP from nine regions within seven countries in a multi-center European study. National policies and legislation, support and health services, assistive technology, and the physical environment are likely to cause these differences (24).

Since participation is a multidimensional construct, a variety of instruments based on the ICF framework have been developed to measure participation in children and adolescents with disabilities (25). The *Children's Assessment of Participation and Enjoyment* (CAPE) has been used to measure participation in recreation and leisure activities in children with and without disabilities, aged between 6 and 21 years (26). CAPE was developed in a longitudinal study in Canada with children with physical disabilities, particularly CP (27), and is a child-friendly self-report instrument that records the behavioral (diversity and intensity of participation), contextual (with whom and where activities are done) and affective (enjoyment) aspects of participation, based on information collected directly from the child (28). CAPE covers core participation constructs, such as objective *(“being there”)* and subjective *(“in the moment*”) elements of experience (29). CAPE content validity was obtained by comprehensive literature review, consulting with experts, and pilot testing (28). Reliability and validity were established using data from a longitudinal study with children with physical disabilities (26). Supportive evidence for the construct validity was obtained from prediction correlation with child and family variables (27, 30), and quality of life domains (31). Test-retest reliability was satisfactory (30–32). A systematic review showed that the CAPE is a valid participation measure for CP children (33). Translated and adapted versions have been validated (30–32, 34, 35), thereby allowing comparisons between countries (22, 36).

Although the prevalence of CP in Portugal is decreasing, it is still the most common physical disability in children (37, 38). A validated Portuguese version of the CAPE would be instrumental in identifying the patterns of leisure participation in this population, in order to design strategies to promote participation and thereby improve child care. Additionally, this information would be instrumental in providing information for health and education professionals, community services, and public policies.

The aim of the present study is to assess the validity and reliability of the CAPE Portuguese (European) version in children with and without CP, aged between 8 and 18 years. Translated versions require analysis to ensure an adequate cultural adaptation and equivalence (39). Thus, construct validity was determined by assessing (i) whether CAPE identifies know-groups differences between children with and without CP, and (ii) there is a significant correlation between participation (diversity, frequency, and enjoyment) and quality of life domains. To assess construct validity, we followed a previous validation study procedure (31), small to moderate correlations between quality of life and participation measured by CAPE are expected. Reliability was assessed by internal consistency, and test-retest reliability, with the hypothesis that our results are similar to the original study and translated versions.

## Materials and Methods

### Participants

**Figure 1:**
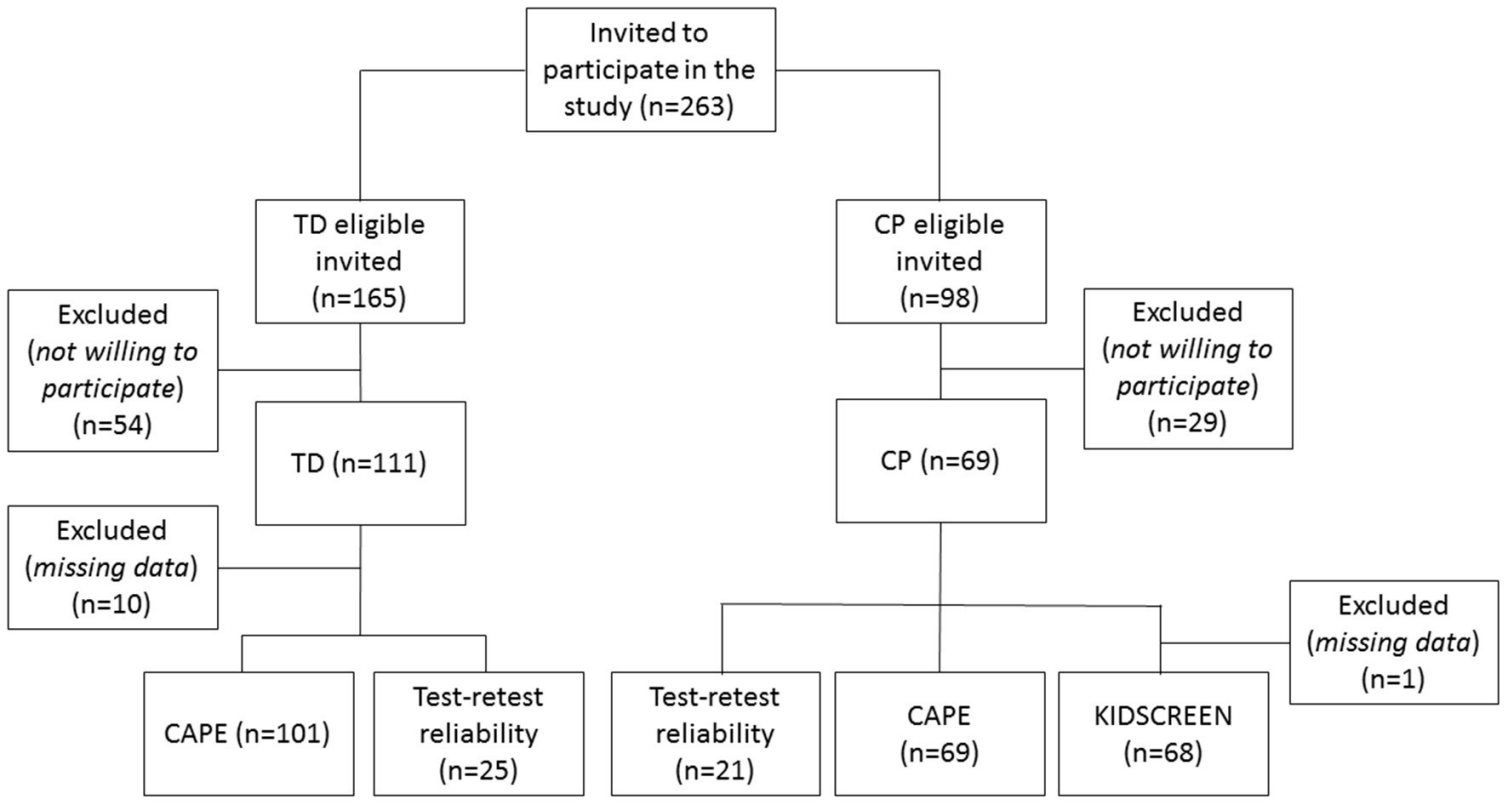
Flow chart of the recruitment for the study.

After initial invitation sent to 263 children (Figure 1), a convenience sample of 170 children and adolescents (mean age = 12.5 years; SD = 2.91; range 8–18 years) with CP (*n* = 69) or typical development (TD, *n* = 101) participated in the study (Table 1).

**Table 1:** Sample characteristics.

		**Children with CP** **(*n* = 69)** ***n* (%)**	**Children with TD** **(*n* = 101)** ***n* (%)**	**All sample** **(*n* = 170)** ***n* (%)**	***p-*value**
Gender	Male	45 (65.2)	50 (49.5)	95 (55.9)	*p* = 0.041
	Female	24 (34.8)	51 (50.5)	75 (44.1)	
Age (years)		Mean = 12.75 (SD = 2.95)	Mean = 12.44 (SD = 2.89)	Mean = 12.5 (SD = 2.91)	
	8–12	31 (44.9)	51 (50.5)	82 (48.2)	*p* = 0.468
	13–18	38 (55.1)	50 (49.5)	88 (51.8)	
Intellectual Disability	None	14 (20.3)	–	–	
	Mild	31 (44.9)	–	–	
	Moderate	24 (34.8)	–	–	
GMFCS	Level I	33 (47.8)	–	–	
	Level II	11 (15.9)	–	–	
	Level III	12 (17.4)	–	–	
	Level IV	7 (10.1)	–	–	
	Level V	6 (8.7)	–	–	

*CP, Cerebral palsy; TD, Typical development; GMFCS, Gross Motor Function System*.

Participants with CP were identified and contacted by the rehabilitation services of five pediatric rehabilitation centers in the Lisbon area and South of Portugal. Parents of children with CP were invited by telephone or directly at the rehabilitation service, and the objectives and procedures of the study were explained. For those who agreed to participate, an interview was scheduled according to the availability of the family. Participants were given the opportunity to choose the assessment site, either at home or at the rehabilitation service, to reduce interference with the family routine to a minimum. The inclusion criterion was having a medical diagnosis of cerebral palsy. Exclusion criteria were having a severe intellectual disability, a botulinum toxin injection in the last 6 months, or orthopedic surgical intervention in the last 12 months. The group means age was 12.75 years (SD = 2.95) and included 45 males and 24 females with different levels in the Gross Motor Function Classification System (GMFCS) (level I: 33; level II–III: 23; level IV–V: 13).

Participants with TD were recruited from three regular public schools in the Center of Portugal. Parents received an invitation letter with the study explanation and consent form. The inclusion criteria were the absence of disability and orthopedic surgical intervention in the last 12 months. Eligible participants were identified by the teacher. In this group, the mean age was 12.44 (SD = 2.89) and included 50 males and 51 females.

This study was carried out in accordance with the recommendations of the Ethics Board of the Faculty of Human Kinetics (University of Lisbon). All parents of the subjects gave written informed consent in accordance with the Declaration of Helsinki and all subjects (children) gave verbal or written assent prior to data collection. The protocol was also approved by the Ethics Commission of Centro de Medicina de Reabilitação do Alcoitão.

### Instruments

#### Children's Assessment of Participation and Enjoyment

CAPE is a self-report measure of participation in 55 recreation and leisure activities, to assess children and youth between 6 and 21 years with and without disabilities. CAPE can be administered in questionnaire or assisted-interview formats, either with or without parental assistance (26).

CAPE provides information on five dimensions of participation over the previous 4 months: *Diversity* (Have you done this activity in the past 4 months? 1 “yes”/0 “no”), if yes, the participant answers the following questions about *Intensity* (How often have you done this activity? from 1–“one time in the past 4 months” to 7–“one time a day or more”), *With Whom* (With whom have you done this activity most often? from 1–“alone” to 7–“with others—i.e., coaches, teachers, tutors”), *Where* (Where have you done this activity most often? from 1–“at home” to 6–“beyond your community”), *Enjoyment* (How much do you like or enjoy doing this activity? from 1–“not at all” to 5–“love it”) (26).

Each of these five dimensions offers three levels of scoring: (I) overall participation score (55 items); (II) domain scores: formal (structured activities that involve rules or goals, typically conducted by a coach or instructor, 15 items), and informal (activities with little or no prior planning often initiated by the child, 40 items); (III) activity type scores: recreational (12 items: e.g., playing board or card games, watching tv), physical (13 items: e.g., bicycling, doing team sports), social (10 items: e.g., going to a party, visiting friends), skill-based (10 items: e.g., learning to sing, playing a musical instrument), and self-improvement (10 items: e.g., doing a religious activity, reading) (28).

#### KIDSCREEN-52 Parent Version

The *KIDSCREEN* is a 52-item generic health-related quality of life measure to healthy and chronically ill children and adolescents aged 8–18 years and is designed as a child or parent report. This cross-cultural and standardized instrument was developed based on literature review, expert consultation, and focus groups across Europe (40). *KIDSCREEN-52* has shown satisfactory psychometric properties (41, 42).

*KIDSCREEN* assesses 10 domains of quality of life: physical well-being, psychological well-being, moods and emotions, self-perception, autonomy, parental relations, financial resources, social support and peers, school environment, and social acceptance. For each domain, the relevant items are summed and scaled to yield a score in the range 0–100 with higher scores indicating a higher quality of life.

#### Gross Motor Classification Function System (GMFCS)

GMFCS is a valid and reliable 5-level classification system that describes the gross motor function of children CP based on their self-initiated movement. The general headings range from level I (walks without limitations) to level V (transported in a manual wheelchair) (43).

#### Socio-Demographic Profile

Information on gender, age, and intellectual disability was recorded according to the participant's clinical information. For intellectual disability, none or mild (IQ ≥ 70) and moderate (50> IQ <70) were considered.

### Procedures

#### Translation

Permission to translate the CAPE was obtained from the publisher Pearson Corporation. The translation and cultural adaptation were performed in five stages (39, 44). The original version was translated by three Portuguese-native translators. A synthesis of the translations was carried out by the research team, including an expert in the Portuguese language. The pre-version was reverse-translated by two professional English native translators. An expert panel comprised by eight researchers with complementary expertise (validation of scales, rehabilitation, and special education) and a parent of a child with CP assessed the content validity by evaluating semantic, idiomatic, experimental, and conceptual adequacy. The experts were instructed to express disagreement with item description and to evaluate the suitability of the construct. Participants scored each item according to relevance using a 4-point Likert scale ranging from “highly relevant” (score 4), “quite relevant” (score 3), “somewhat relevant” (score 2), to “not relevant” (score 1). We calculated whether CAPE Portuguese version has an appropriate sample of items for the construct by using the overall Content Validity Index (S-CVI/Ave) and the Individual Item Content Validity Index (I-CVI). For this calculation, the number of ratings on relevant scores (scores 3 and 4) was summed and divided by the number of evaluators for each item (I-CVI). For S-CVI/Ave, the I-CVI average of all scale items was calculated. Reference values for excellent content validity index were S-CVI/Ave (>0.90) e I-CVI (>0.78) (45).

After discussion, the S-CVI/Ave calculated for the CAPE Portuguese version was 0.93. One item (“doing a paid job”) received a low I-CVI score (0.63) and generated a discussion regarding its suitability for the study's proposed age group. The item was maintained in the Portuguese version not only because youth engagement in occasional paid jobs is sometimes observed, but also because consistency with the other CAPE versions is necessary to allow cross-culture comparisons.

A convenience sample (*n* = 16) was formed to carry out the pre-test of the CAPE Portuguese version (8 CP children; 8 TD children; 8–18 years). The CP group included children with different levels of gross motor function (level I: 3; level II–III: 2; level IV–V: 3), including a child using an augmentative and alternative communication device (computer with vocalizer). Participants reported understanding the guidelines, items, and response options. No activity had to be added or excluded. The final version was accepted by CAPE copyright holders.

#### Assessment

After consent form assignment, parents of children with CP answered the *KIDSCREEN*. Participants with CP responded to the CAPE by assisted-interview accompanied by a parent. Assisted-interview was the chosen method because it is more enjoyable for the participant, and minimizes the influence of physical impairments on manual completion assessment. Following manual instructions (28), parents were asked to allow the answers to be given by the children, assisting them if necessary but not answering for them. In all situations the child was encouraged to answer independently. The assessments were conducted by the first author (FV-N). Gross motor function, intellectual disability and socio-demographic information were obtained from the clinical data available in the rehabilitation services. Participants with TD answered the questionnaire in class, after guidance on the questionnaire items and response options. To perform retest, 46 participants (21 with CP; 25 with TD) responded to the CAPE twice within a two-week interval at the same conditions.

### Statistical Analysis

Descriptive statistics were used to characterize the sample, diversity, and intensity scores. Diversity refers to the number of different activities and intensity to the frequency of activities carried out. For the CAPE, mean scores were calculated when at least 80% of the items were completed (28).

The known-groups method was used to support Portuguese CAPE construct validity by determining whether the test scores discriminate across groups that are theoretically known to differ. To assess differences in diversity and frequency scores between children with and without CP, independent sample *t*-tests were performed. After the Bonferroni adjustment, the significance level for the *t*-tests was set at *p* < 0.004. The Pearson-product moment was used to assess the correlation between overall diversity, frequency and enjoyment scores, and *KIDSCREEN* domains in the group of children with CP. The magnitude of the correlations will be assessed according to the effect size proposed by Cohen (46), small (*r* = 0.1), moderate (*r* = 0.3) and large (*r* = 0.5), with *p* < 0.01. *KIDSCREEN* scores were analyzed when missing data did not exceed one item in each domain (40).

The internal consistency of the overall, formal and informal, and activity types frequency scores were examined by Cronbach's alpha for the entire sample. Alpha coefficients between 0.70 and 0.95 were considered good (47).

Test-retest reliability was expressed by Intraclass Correlation Coefficients (ICC; two-way mixed effects model; absolute agreement) as a ratio between 0 and 1. Good reliability was considered for ICC values equal or higher than 0.70 (47). Standard Error of Measurement (SEM) and Smallest Detectable Change (SDC) were also calculated. SEM equals the square root of the error variance, and SDC reflects the smallest within-person change in the score which, with *p* < 0.05, can be interpreted as a real change above measurement error (SDC = 1.96 × √2 × SEM) (48). Statistical analyses were performed using the SPSS 24.0 version software program.

## Results

One hundred and eighty children participated in the study. However, 10 CAPE questionnaires were excluded due to missing data. Thus, 170 children, 69 with CP (40.6%) and 101 with TD (59.4%), were included in the analysis.

Table 2 shows the means of participation diversity by formal and informal domains and activity type. Children with CP have low participation diversity in all activity types, with statistical significance in recreational [t_(168)_ = 3.54, *p* = 0.001], active physical [(t_(168)_ = 3.23, *p* = 0.001], social [t_(168)_ = 2.94, *p* = 0.002], and self-improvement activities [t_(168)_ = 8.13, *p* < 0.001]. Although there were differences in skill-based activities score means, they were not statistically significant [t_(168)_ = 0.54, *p* = 0.586]. Low participation diversity in the CP group was also identified in formal [t_(167)_ = 3.03, *p* = 0.003] and informal domains [t_(168)_ = 5.66, *p* < 0.001].

**Table 2:** Comparison of CAPE diversity scores between CP and TD children.

**Domains and activity types (range)**	**Children with CP** **(*N* = 69)** **M (SD)**	**Children with TD** **(*N* = 101)** **M (SD)**	***T-test***	***p-value***
Formal domain (0–15)	1.9 (1.3)	2.6 (2.0)	−3.036	*p* = 0.003
Informal domain (0–40)	19.0 (4.9)	23.7 (5.6)	−5.661	*p* < 0.001
Recreational activities (0–12)	6.5 (2.0)	7.7 (2.4)	−3.548	*p* = 0.001
Active physical activities (0–13)	2.3 (1.6)	3.1 (1.7)	−3.233	*p* = 0.001
Social activities (0–10)	6.9 (1.6)	7.6 (1.6)	−2.943	*p* = 0.002
Skill-based activities (0–10)	1.8 (1.0)	1.9 (1.6)	−0.545	*p* = 0.586
Self-improvement activities (0–10)	3.4 (2.0)	6.0 (2.0)	−8.134	*p* < 0.001

*Bonferroni adjustment of the significance level was set at p < 0.004. TD, Typical development; CP, Cerebral palsy*.

Table 3 shows the mean of participation frequency on formal and informal domains and activity type. Children with CP have low participation frequency in all activity types, with statistical significance in active physical [t_(168)_ = 3.64, *p* < 0.001]; social [t_(168)_ = 3.71, *p* < 0.001]; and self-improvement activities [t_(168)_ = 7.63, *p* < 0.001]. Recreational [t_(160)_ = 1.25, *p* = 0.215] and skill-based activities [t_(167)_ = 0.81, *p* = 0.418] intensity scores differences did not show statistical significance. The CP group also presented low participation frequency in formal [t_(165)_ = 3.46, *p* = 0.001] and informal domains [t_(168)_ = 5.07, *p* < 0.001].

**Table 3:** Comparison of CAPE frequency scores between CP and TD children.

**Domains and activity types (range 0–7)**	**Children with CP** **(*N* = 69)** **M (SD)**	**Children with TD** **(*N* = 101)** **M (SD)**	***T-*test**	***p-*value**
Formal domain	0.6 (0.4)	0.9 (0.7)	−3.036	*p* = 0.001
Informal domain	2.2 (0.5)	2.7 (0.7)	−5.073	*p* < 0.001
Recreational activities	2.8 (0.9)	3.0 (1.0)	−1.245	*p* = 0.215
Active physical activities	0.8 (0.6)	1.1 (0.7)	−3.642	*p* < 0.001
Social activities	2.9 (0.7)	3.4 (0.9)	−3.708	*p* < 0.001
Skill-based activities	0.8 (0.5)	0.9 (0.8)	−0.812	*p* = 0.418
Self-improvement activities	1.6 (1.0)	2.8 (1.0)	−7.630	*p* < 0.001

*Bonferroni adjustment of the significance level was set at p < 0.004. TD, Typical development; CP, Cerebral palsy*.

One *KIDSCREEN* questionnaire had missing data and was therefore excluded. Small to moderate correlations were found between physical well-being and diversity [r_(66)_ = 0.289, *p* = 0.017]; psychological well-being and frequency [r_(66)_ = 0.280, *p* = 0.021] and enjoyment [r_(66)_ = 0.264; *p* = 0.030]; and diversity and the school domain [r_(66)_ = −0.249, p = 0.040] (Table 4). The correlation between physical well-being and frequency of participation was statistically significant [r_(66)_ = 0.336, *p* = 0.005].

**Table 4:** Correlation coefficients between CAPE and KIDSCREEN-52 domains.

**KIDSCREEN domains/ CAPE dimensions**	**Children with CP (*****n*** **= 68)**		
	**Diversity**	**Frequency**	**Enjoyment**
Physical well-being	0.289^*^	0.336^**^	0.019
Psychological well-being	0.209	0.280^*^	0.264^*^
Mood and emotions	−0.120	−0.042	0.028
Self-perception	−0.014	0.092	−0.058
Autonomy	0.055	0.111	−0.057
Parent relations and home life	−0.152	−0.059	−0.065
Financial resources	0.218	0.176	−0.180
Social support and peers	0.007	0.053	−0.064
School environment	−0.249^*^	−0.160	0.130
Social acceptance	−0.196	−0.074	−0.021

* p < 0,05;

** *p < 0,01. CP, Cerebral Palsy*.

Reliability analysis was performed on the overall scale, formal and informal domains, and activity type frequency scores. Alpha values showed good internal consistency for the overall scale (0.79) and formal domains (0.75). The alpha values were low for the informal domain (0.46) and activity types: recreational (0.50), social (0.47), active physical (0.48), skill based (0.40), and self-improvement (0.65).

Test-retest reliability correlation coefficients were good, ranging between 0.74 and 0.83 in the CP group and between 0.82 and 0.91 in the TD group (Table 5). The SDCs ranged between 1.27 and 2.75 in the CP group, and between 1.55 and 3.06 in the TD group.

**Table 5:** Test-retest reliability of CAPE frequency scores.

	**Children with CP** **(*****n*** **= 21)**				**Children with TD** **(*****n*** **= 25)**			
	**ICC**	**CI (95%)**	**SEM**	**SDC**	**ICC**	**CI (95%)**	**SEM**	**SDC**
Formal	0.91	0.79–0.96	0.46	1.32	0.93	0.86–0.95	0.62	1.72
Informal	0.86	0.68–0.94	0.56	1.55	0.90	0.83–0.94	0.65	1.81
Recreational	0.80	0.58–0.91	0.99	2.75	0.86	0.77–0.92	1.10	3.06
Physical	0.79	0.55–0.90	0.60	1.67	0.83	0.71–0.90	0.66	1.84
Social	0.74	0.44–0.88	0.76	2.09	0.82	0.70–0.90	0.76	2.11
Skill-based	0.83	0.63–0.93	0.55	1.52	0.91	0.84–0.95	0.72	2.00
Self-improvement	0.81	0.59–0.92	0.77	2.13	0.89	0.81–0.94	1.00	2.77

*Two-way mixed effects model; absolute agreement; ICC, Intraclass correlation coefficient; CI, Confidence interval; SEM, Standard error of measurement; SDC, Smallest detectable change*.

## Discussion

The aim of this study was to assess validity and reliability of the CAPE Portuguese version. The assessment of participation is critical for follow-up monitoring and intervention in children with CP, as participation is an important outcome in pediatric rehabilitation (49, 50). Leisure provides opportunities for child development and for improving physical and psychosocial well-being (15). The validation of a measure conceptually grounded on ICF provides relevant information about the involvement of children in everyday life situations, allowing discussion and comparison of results between different countries (1).

### Construct Validation

To further investigate CAPE construct validity, we tested the hypothesis that there are significant differences between the participation scores of children with and without disabilities. Our data revealed that Portuguese children with CP have lower diversity and frequency of leisure participation than children with typical development (TD). Participation diversity was low in formal (structured, planned activities) and informal (non-structured, self-initiated activities) domains, and recreational, active physical, social, and self-improvement activities. Furthermore, children with CP showed a low frequency of participation in formal and informal domains, and active physical, social, and self-improvement activities.

In agreement with our study, differences in participation between groups of children with and without disabilities were observed in CAPE studies conducted in other countries. A study with 398 Spanish children with and without CP (31) identified lower diversity for the CP group in both formal and informal domains, recreational, active physical and self-improvement activities. Bult et al. (30) also found significant differences between Dutch children with TD and those with physical disabilities, including CP, in the frequency of participation in physical, social, self-improvement and recreational activities. These findings are consistent with previous research (9, 14, 16, 23).

Our findings showing a positive correlation between leisure participation and quality of life in children with CP also support construct validity. We found that frequency of participation in leisure activities is positively related to quality of life in the physical well-being domain in children with CP, which is in agreement with previous research (9, 10, 51). Dahan-Oliel et al. (10) identified a relationship between participation in leisure activities and different dimensions of quality of life in children with neuro-disabilities. Furthermore, McManus et al. (9) showed that overall participation in everyday activities has a significant effect on the quality of life of Irish school-aged children with CP. In that study, a one-unit increase in participation frequency was associated with a 7.8 units increase in the quality of life related to physical well-being. Shikako-Thomas et al. (51) suggests that children who participate in more leisure activities may experience better quality of life than children who participate in fewer or less frequent activities. The authors also argue that the direction of causality may be reverse, such that children who experience a higher sense of physical and psychosocial well-being may engage in more leisure activities.

Interestingly, Longo et al. (31) also found positive correlations between leisure participation and other domains of quality of life, such as psychological well-being, financial resources, autonomy, and social support and peers. The differences between the present study and Longo's study may be due to differences between samples. In Longo's et al study (31), some participants were children with CP with severe intellectual disability, whereas in our study participants with severe intellectual disability were not included. Indeed, Arnaud et al. (52), based on parental reports, found that the severity of intellectual impairment in children with CP was strongly associated with low quality of life in physical well-being, autonomy, and social support domains, suggesting that these children are less able of creating social time or maintaining relationships with their peers.

### Reliability

Reliability of CAPE was not entirely satisfactory. The internal consistency of the CAPE Portuguese version is considered good (0.80) for the overall scale. The results for formal (0.46) and informal domain (0.75), and activity types (0.40 to 0.65) are similar to those identified in the original study, where formal and informal alpha values were 0.42 and 0.76, respectively, and activity types ranged from 0.32 to 0.62 (28). The authors argued that low alpha values are expected since frequency scores are determined by various environmental, family, and child factors. Thus, our results are in agreement with the findings of other versions (32, 34, 35). Regarding the test-retest analysis, our ICC results were adequate, superior to 0.74 in the group with CP, and 0.82 in the TD group, in formal and informal domains, and activity types. SDC values indicate that in both groups, changes would have to be significant for recreational activities to be considered beyond the measurement error.

### Limitations and Future Directions

CAPE is a child-friendly measure that allows the inclusion of participants with different abilities since it uses figures of the activities and can be answered with assistance. In spite of that is an extensive scale, and in some situations, the time to answer it may be too long. Participants took an average of 40 min to respond to CAPE; however, some participants needed more time (i.e., a child using assistive technology for communication). In these cases, it may be difficult for the child to sustain attention and concentration. Moreover, the 4-month recall period appears to be lengthy, mainly to respond to less frequent activities.

This study has limitations, such as the use of a sample of children with CP, which excluded children with severe intellectual impairment, and those who underwent interventions that temporarily influenced activity and family routine. Another limitation might be that parental assistance was available, if necessary, only to the participants with CP. Correlations between quality of life and participation were found in previous studies with children with neurodisabilities (9, 10). However, future studies including children without disabilities, may reinforce the relationship between these constructs. In the present study, children's preference was not assessed, but its relationship with participation has been previously established (32, 35), and could be explored in the future in a Portuguese sample. Quality of life was assessed only from the parents' perspective, although differences between the child' and parent's reports could be expected. Although participation refers to the interaction between person and environment in everyday life situations, we did not analyze how environmental factors influence leisure participation, being also an important approach for future studies. Finally, this study still does not provide normative data of CAPE for Portuguese children. Future studies with larger samples should address this issue.

## Conclusion

CAPE is a comprehensive measure that enables identification of leisure participation patterns for clinical and research purposes, covering subjective and objective elements of experience. Also, it can provide relevant information for health and education professionals who assist children with CP. Our results show that the CAPE Portuguese (European) version has adequate construct validity and test-retest reliability to assess children with and without CP aged from 8 to 18 years.

## Data Availability Statement

The raw data supporting the conclusions of this manuscript will be made available by the authors, without undue reservation, to any qualified researcher.

## Author Contributions

FV-N, RC, and RO: conceived and designed the study and wrote the paper; FV-N: performed the experiments; FV-N and RC: analyzed the data.

### Conflict of Interest Statement

The authors declare that the research was conducted in the absence of any commercial or financial relationships that could be construed as a potential conflict of interest.
