# Corrigendum: Cross-Cultural Validation of Children's Assessment of Participation and Enjoyment Portuguese Version

**DOI:** 10.3389/fped.2019.00167

**Published:** 2019-04-30

**Authors:** Fábio Vila-Nova, Raul Oliveira, Rita Cordovil

**Affiliations:** ^1^Faculdade de Motricidade Humana, Universidade de Lisboa, Lisbon, Portugal; ^2^Centro Interdisciplinar da Performance Humana, Faculdade de Motricidade Humana, Universidade de Lisboa, Lisbon, Portugal

**Keywords:** participation, leisure, children, CAPE, measure, validation, cerebral palsy

There is an error in the Funding statement. The correct number for the Foundation for Science and Technology (Portugal) I&D 472 is (UID/DTP/00447/2019). The authors apologize for this error and state that this does not change the scientific conclusions of the article in any way. The original article has been updated.

